# Climate change impacts on potential recruitment in an ecosystem engineer

**DOI:** 10.1002/ece3.419

**Published:** 2013-02-04

**Authors:** Emer Morgan, Ruth M O' Riordan, Sarah C Culloty

**Affiliations:** Aquaculture and Fisheries Development Centre, School of Biological, Earth and Environmental Sciences, University College CorkIreland

**Keywords:** *Cerastoderma edule*, climate variability, condition index, ecosystem engineer, gametogenesis, phenology

## Abstract

Climate variability and the rapid warming of seas undoubtedly have huge ramifications for biological processes such as reproduction. As such, gametogenesis and spawning were investigated at two sites over 200 km apart on the south coast of Ireland in an ecosystem engineer, the common cockle, *Cerastoderma edule*. Both sites are classed as Special Areas of Conservation (SACs), but are of different water quality. *Cerastoderma edule* plays a significant biological role by recycling nutrients and affecting sediment structure, with impacts upon assemblage biomass and functional diversity. It plays a key role in food webs, being a common foodstuff for a number of marine birds including the oystercatcher. Both before and during the study (early 2010–mid 2011), Ireland experienced its two coldest winters for 50 years. As the research demonstrated only slight variation in the spawning period between sites, despite site differences in water and environmental quality, temperature and variable climatic conditions were the dominant factor controlling gametogenesis. The most significant finding was that the spawning period in the cockle extended over a greater number of months compared with previous studies and that gametogenesis commenced over winter rather than in spring. Extremely cold winters may impact on the cockle by accelerating and extending the onset and development of gametogenesis. Whether this impact is positive or negative would depend on the associated events occurring on which the cockle depends, that is, presence of primary producers and spring blooms, which would facilitate conversion of this extended gametogenesis into successful recruitment.

## Introduction

It is evident that changes are ongoing in the marine environment in terms of temperature, wind patterns, and sea levels orchestrated by climate change, with the swift nature of such changes causing much concern (Philippart et al. 2011). These changes are being seen globally, but not equally and the seas around Europe are disparately affected and were included as “rapid warming” seas in recent studies of warming in large marine ecosystems (Belkin 2009; Philippart et al. 2011). The period 1965–2004 in the North Atlantic has been a time of net heat accumulation and yields from European fisheries have also shown changes, with decreases in the Celtic Biscay Sea area, but increases in the Norwegian Seas (Philippart et al. 2011). Trends in the Irish Sea indicate warming of sea surface temperatures by 0.5–1°C from 1870 to 2007, as well as a tendency for increases in air temperature, sea level, and acidification (UKMMAS 2010). The sea surface temperature around the United Kingdom and Ireland has increased at six times the rate of the global average (Frost et al. 2012). For the preceding 30 years, there have been increased of 0.2–0.6°C in sea temperature per decade around the United Kingdom and Ireland, and the mean surface ocean pH has reached 8.14 in comparison with 8.25 in the 18th century (Heath et al. 2012). The mean annual air temperature recorded in Ireland has increased by 0.7°C in the period 1890–2004 and the northern hemisphere has been warmer since 1980 than at any time in the last 2000 years (Philippart et al. 2011). However, in 2010, Ireland experienced its coldest winter in 50 years (http://www.met.ie/climate-ireland/major-events.asp), related to the negative phase of the North Atlantic Oscillation (NAO), whose variability could be related to anthropogenic warming (Goodkin et al. 2008). It is predicted that sea levels around Ireland will increase by 18–59 cm in the 21st century; water quality is expected to be impacted deleteriously by the re-entry of contaminants that had previously been sediment-bound in lowlands and estuaries during extreme events that generate run off (Schiedek et al. 2007). As a result, species which have small thermal tolerance windows will be jeopardized (http://www.epa.ie/downloads/pubs/climatechange/EPAandClimateChangeFinal.pdf).

**Figure 1:**
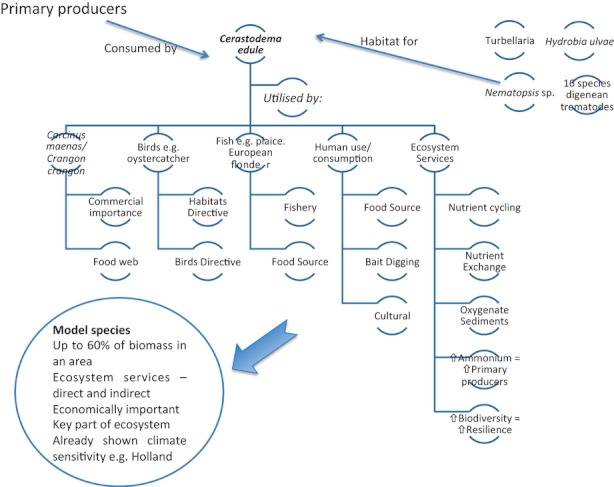
Simplified diagram of ecosystem including services of cockles in its habitats showing its appropriateness as a model species for climate variability studies.

*Cerastoderma edule* is a common infaunal bivalve whose range spans from the Barents Sea to West Africa and is less commonly found in the southwest Mediterranean (Reise 2003; Cardoso et al. 2009). Preferred habitats of *C. edule* include sandy bays and estuaries, but it can also be found in gravel and mud (http://www.fao.org/fishery/species/3535/en; André et al. 1999; Reise 2003). Cockles can comprise up to 60% of the biomass of an area (Dabouineau and Ponsero 2009) and they play an important role in the ecosystem (Fig. 1). They are a link between primary producers (plankton) and higher trophic levels as cockles are predated upon by crabs, for example, *Carcinus maenas*, shrimp, for example, *Crangon crangon*, fish, for example, *Pleuronectes platessa*, and birds, for example, *Haematopus ostralegus*. Indeed *C. crangon* and *P. platessa* may consume up to 90% of juvenile cockles in an area (Cesar 2009). *Carcinus maenas*, *C. crangon*, and *P. platessa* are themselves subject to fisheries, as is the cockle and therefore create a link between marine and terrestrial food webs, in addition to being commercially important. Cockles themselves provide a habitat for up to 16 species of digenean trematode (de Montaudouin et al. 2009), turbellaria, and various other parasites, acting as a first and second intermediate host. Cockles too are responsible for a range of other ecosystem services, such as recycling nutrients, acting as a carbon store, cycling energy, and increasing ammonium concentrations, which in turn increases primary producers. They add to the overall diversity of an area and it is thought that increased biodiversity may increase ecosystem resilience and by their movements in the sediment they facilitate the exchange of dissolved nutrients to the water column and may increase the depth to which oxygen dissipates into the sediment (Cesar 2009). It has been suggested that estuarine and coastal areas are prime candidates for the study of climate change as they are shallow, and alterations in thermal regimes will be readily apparent (Moore et al. 2011; Madeira et al. 2012). Therefore, cockles were identified as an ideal candidate to evaluate the effects of climate variability on biological processes and in the case of this study: reproduction.

Previous research has shown that recruitment in cockles can vary markedly in success between years and sites, with large increases in density occurring over a short period of time (Ramόn 1996; Honkoop et al. 1998; Sauriau and Kang 2000; Cardoso et al. 2009). Although cockles are known to be sensitive to colder conditions, experiencing mortalities during cold spells (Ramόn 1996; Strasser et al. 2001; Flach 2003; Reise 2003; Tyler-Walters 2007), greater recruitment has been seen in many areas, for example, Burry Inlet, following severe winters (Yankson 1986; André and Rosenberg 1991; Lindegarth et al. 1995). A decline in recruitment among *C. edule* and other bivalves (*Mytilus edulis*, *Mya arenaria*, *Macoma balthica*) in the Dutch Wadden Sea and Sweden has been linked to warming winters for the previous 15 years (Cardoso et al. 2009). It has been shown that following warmer winters, smaller, less viable eggs, with less lipids, are produced (Cesar 2009) and gametogenesis can extend further in the year and not allow for a sufficient rest period for recovery, prior to the beginning of the next reproductive cycle (Guillou and Tartu 1992). If conditions for optimal spawning (cold winters) are rarer, short lived species, such as the cockle, may not experience them in their life time (Cardoso et al. 2009). This could lead to an overall decrease in cockle biomass, which would have serious ramifications for not only cockles, but the ecosystem as a whole, because they as previously stated are an integral part of their habitats.

*Cerastoderma edule* is a dioecious, broadcast spawner (Boyden 1971; Dabouineau and Ponsero 2009); therefore, fertilization in cockles is external with males and females producing gametes, which are released into the water column (Lindegarth et al. 1995; André et al. 1999). It is an income breeder, acquiring energy as it produces gonads. Growth of cockle planktonic larvae proceeds quickly, and they persist in the water column for at least 2–3 weeks, but perhaps up to 5 weeks (Boyden 1971; Lindegarth et al. 1995; Honkoop and van der Meer 1998; André et al. 1999; Bouma et al. 2001; Reise 2003). Sexual maturity is usually reached when cockles are 18 months old or of shell length of 15–20 mm (Seed and Brown 1977; Dabouineau and Ponsero 2009), but Cardoso et al. (2009) recorded gonads in cockles >12 mm in shell length in cockles from the Dutch Wadden Sea. Settlement of cockles at Strangford Lough, Ireland has been described in August and September (Seed and Brown 1977), but in France between September and October (Guillou and Tartu 1994). External parameters such as temperature are known to affect gametogenesis and onset of spawning in bivalves (Gosling 2003; Drummond et al. 2006). Temperature acts to synchronize internal processes in bivalves, such as nutrient storage and the endocrine system (Brown 1984).

The aims of this study were to examine the reproductive cycle of *C. edule,* using it as a model species to assess the effects of climate variability on an a *r* – selected, ecosystem engineer, which is integral to food webs, provides habitat for other organisms and has commercial importance. The study was undertaken at two sites, which are classed as Special Areas of Conservation – SACs which are areas created under Article 3 of the EU Habitats Directive, and their purpose is to aid in the conservation of species and habitats on Annex I and Annex II of the Habitats Directive, by having conservation sites of good quality (http://www.epa.ie/licences/lic_eDMS/090151b2803baf8e.pdf; http://www.epa.ie/downloads/pubs/water/waterqua/waterrep/; http://jncc.defra.gov.uk/page-23). Two sites were chosen as great variation can be seen in cockles separated by a few meters on the same bed – so this study aimed to determine whether there was a general pattern apparent in reproduction (e.g., initiation of gametogenesis/spawning), despite local variation (e.g., in temperature, water quality) by examining two sites at the same latitude, but 215 km apart. Cockle reproduction has been assessed previously, but rarely using histology, the previous study performed in Ireland was in 1977, and the last study published from Europe was in 1989 and used gamete volume fraction (GVF). So, a histological study was deemed appropriate, which, although labor intensive, gives a much clearer picture than just assessing presence of larvae in the plankton, etc. The alterations in Ireland's weather patterns and climate, due to possible climate change, were especially apparent during this study with two extremely cold winters, the coldest in 50 years (http://www.epa.ie/downloads/pubs/water/waterqua/waterrep/); therefore, this offered a unique opportunity to evaluate the effects of an unusually cold winter on gametogenesis and recruitment in the cockle after a period of prolonged warming.

## Materials and Methods

### Study sites

**Figure 2:**
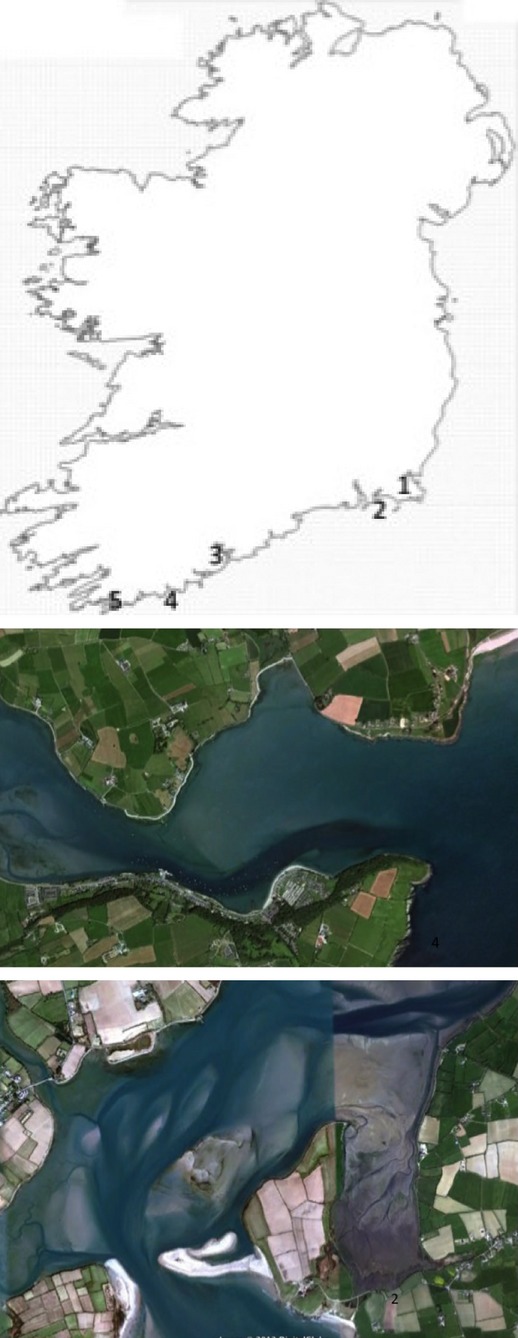
Map of Ireland plus study locations Bannow Bay and Flaxfort Strand with additional temperature stations included (aerial photos taken at 4000 m). 1, Johnstown Castle; 2, Bannow Bay; 3, Cork Airport; 4, Flaxfort Strand; 5, Bantry Bay.

Two sites 215 km apart were selected. Flaxfort Strand (N51°38′ 47.4″, W 008°41′45.0″) is a small semiexposed bay on the southwest coast of Ireland (Fig. 2). It is composed primarily of sand flats and extensive colonization by green algae of *Enteromorpha* spp. can be seen on the upper shore during the summer months. The tidal range is 4.2 m. The River Argideen drains into the area and the water quality is classed as eutrophic by the Environmental Protection Agency (http://www.epa.ie/downloads/pubs/water/waterqua/waterrep/). Land usage of the surrounding areas is predominantly dairy farming. Flaxfort Strand is a part of Courtmacsherry Bay, which is designated as a Special Area of Conservation (SAC: 001230). It was allocated that this status as 10 habitats recorded on Annex 1 of the EU Habitats Directive (http://www.npws.ie/en/SAC/001230/) make up this bay. Clonakilty Bay which is adjacent is classed as a Special Protected Area (SPA: 004081); it includes habitats classed as Annex 1 on the EU Habitats Directive (http://www.npws.ie/en/SPA/004081/) plus bird species with Annex 1 status on the EU Birds Directive, for example, bar-tailed godwits and golden plovers frequent this area.

Bannow Bay (N52°13′07.9″, W006°46′37.8″) is located on the southeast coast of Ireland; it is a shallow bay that is over 10 times larger than Flaxfort Strand. It has an area of ∼1050 hectares and at its greatest is 8 km in length and varies between 1 and 3 km wide and had a tidal range of 3.6 m. Water stays in the bay for two tidal cycles and the bay itself is comprised of a mixture of gravel, sand, and mudflats (Cotter et al. 2010). Unlike Flaxfort Strand, large banks of *Entermorpha* spp. were not observed for the duration of the study and according to the National Parks and Wildlife Service, its habitats are of “general good quality” (http://www.epa.ie/licences/lic_eDMS/090151b2803baf8e.pdf). It is classified as a Special Protected Area – Site Code IE 0004033 under the EU Birds Directive as it too provides habitat for several Annex 1 bird species (the golden plover, bar-tailed godwit) and there are two breeding Annex 1 species present, the little tern and the kingfisher. Under the EU Habitats Directive (92/43/EEC), it is classed as a Special Area of Conservation (Site Code IE000697) as it encompasses 11 Annex 1 habitats as decreed by the Habitats Directive (http://www.epa.ie/licences/lic_eDMS/090151b2803baf8e.pdf). Furthermore, it is also named as a National Heritage Area (NHA Site Code 0000697) and subject to the European Communities (Quality of Shellfish Waters) Regulations S.I No. 268 of 2006 European Communities (Quality of Shellfish Waters) Regulations 2006.

### Environmental parameters

One Stowaway®Tidbit™ (Onset Computer Corporation, Bourne, MA) Weatherproof and Waterproof Temperature Logger was placed in a channel of water at each site, adjacent to the main collection area. For the duration of the study, they were continuously submerged within the channel. The temperature was recorded every 16 min from their deployment until the cessation of the study. The data were retrieved using an Optic Base Station/Coupler Kit and analyzed with BoxCar®Pro 4.3 Starter Kit software (Onset Computer Corporation, Bourne, MA). Additional temperature data for the study period were provided for Bannow Bay by Met Éireann from Johnstown Castle (20.9 km away) and for Flaxfort Strand by the Marine Institute from Bantry Bay (distance of 65.5 km) and Met Éireann from Cork airport (40.5 km from Flaxfort Strand) (Fig. 2).

### Sampling of cockles

The same methodology was employed at both sites and field work was carried out at monthly intervals commencing in March 2010 in Bannow Bay and April 2010 in Flaxfort Strand, continuing until June 2011 at both sites. 30 buried cockles (buried to a depth of 20–30 mm in the sediment) were collected monthly from each site at low tide. Cockles in standing water or those that had surfaced were not sampled for this study, only those that remained buried at low tide were included. Due to adverse weather conditions, cockles were not collected from Bannow Bay in December 2010.

### Morphometrics and age structure

The whole weight, shell weight, and tissue weight were weighed to two decimal places (g). The length of each cockle was measured from the umbo to the lip of shell, the width across the widest part of the shell, and the depth to 1 decimal place in mm. Finally, the number of growth rings on each cockle was counted.

### Condition index

The mean condition index (CI) was calculated on a monthly basis. The average wet tissue weight and average wet shell weight were used as per an altered version of Drummond et al. (2006) and Cotter et al. (2010) (as they used dry weights).


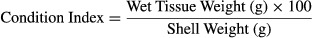


### Histology

In the laboratory, cockles were opened carefully and 10-mm transverse sections through the gonad regions were preserved for histology in Davidson's Solution (Shaw and Battle 1957; Howard and Smith 1983), and refrigerated for 2 days at 4°C. They were sectioned at 5 μm and stained with Hematoxylin and Eosin.

**Figure 3:**
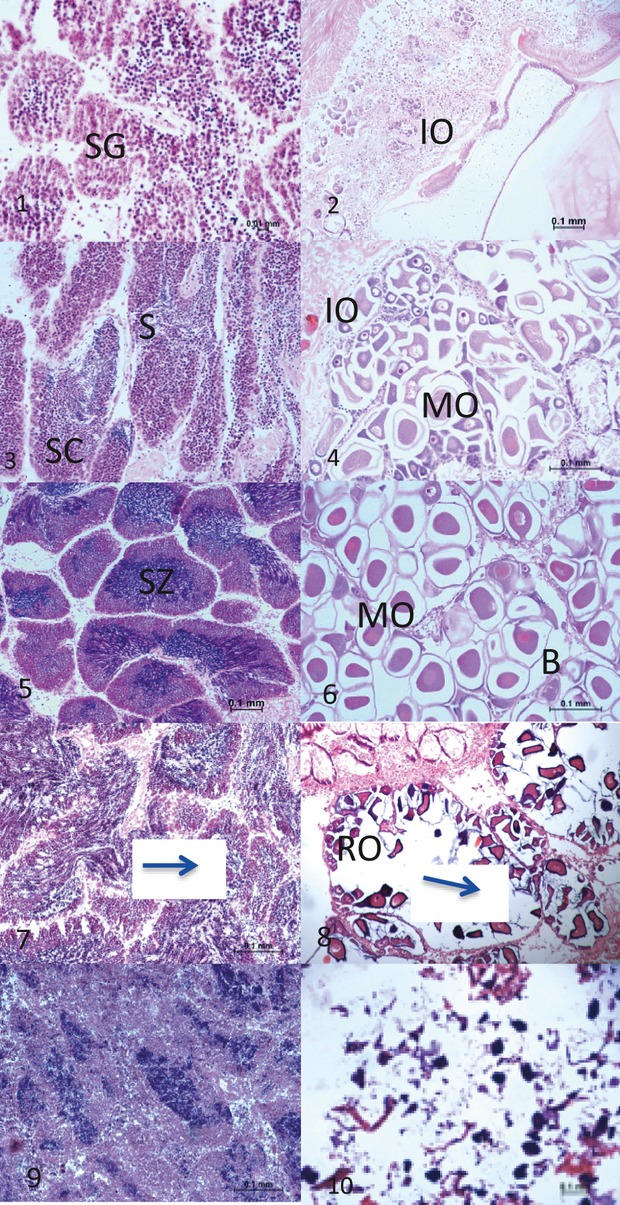
Plates of gonads at each stage of gonadal maturity (taken at 20×, scale bar equals 0.1 mm). (1) Male early developing with spermagonia (SG); (2) female early developing with immature oocytes (IO); (3) male late developing with spermatocytes (SC) and spermatids (S); (4) female late developing with immature oocytes (IO) and a small number of mature oocytes (MO); (5) ripe male with spermatids (S) and spermatozoa (SZ) in plug like arrangement; (6) ripe female mature oocytes (O) and thin barriers (B) between oocytes; (7) spawning partially spent male arrow indicates sperm leaving plugs; (8) spawning partially spent female with residual oocytes (RO) and arrow showing empty spaces in gonads; (9 and 10) spent male and females, respectively.

The slides were examined at 4×, 10×, and 40×. Sex was determined if gonads were present and gonadal maturity stages were assigned to each of the cockles. Five reproductive stages were identified based on a scale from Drummond et al. (2006), which was modified to reflect differences between clam and cockle morphology. These were early developing, late developing, ripe, spawning/partially spent, and spent (Fig. 3, Table 1). When cockles presented stages that could be described as intermediate between two stages, the stage that dominated the greatest part of the gonads was assigned.

**Table 1:** Description of (a) female and (b) male gonads based on a modified scale from Drummond et al. (2006)

Stage	Description
(a)	
Early developing	Small number of oocytes in discrete areas visible with none free in the lumen. Dark in color and with grainy borders.
Late developing	Larger area taken over by oocytes, which have increased in size – still some small in size, with a more defined shape. Some now free from the follicle wall, but remaining in discrete pattern.
Ripe	Majority of the section containing oocytes, which are now more uniformly large. Oval in shape and free in the lumen with thin barriers between oocytes. Appear in distinct net pattern.
Spawning/Partially spent	Broken follicle walls, the number of oocytes reduced. Spaces beginning to show in the gonad region, some oocytes remain that are no longer in a homogenous pattern.
Spent	Section through gonads appears broken, with large spaces evident. Remaining oocytes dark in color and isolated, with no net-like pattern remaining.
(b)	
Early developing	Gonads have begun to develop, small amount discernible in plugs. Comprised mainly of lighter, rounder, and larger spermatogonia encompassing smaller darker spermatocytes. Still appear quite loose in composition.
Late developing	Still mainly spermatogonia and spermatocytes, but some were elongating spermatids visible in the center of the plug. Spermatids stained navy blue in comparison with purple spermatogonia and spermatocytes.
Ripe	Plugs of gonad very organized and intact more compacted than earlier stages. Mainly spermatozoa, tails of the spermatozoa facing the lumen.
Spawning/Partially spent	Spermatozoa leaving plugs, empty spaces visible. The neat organized structure degrading.
Spent	Small amounts of sperm visible scattered through section, follicles broken.

### Statistics

Data were analyzed using MINITAB® Release 14 and PASW Statistics 17 statistical software. Normality was determined using a Kolmogorov–Smirnov Test. A Mann–Whitney test was used to determine if there was a significant difference between the mean temperature; mean length; mean whole, tissue, and shell weights; or CI between the sites. A Kruskal–Wallis nonparametric test was used to assess if there was a significant difference between monthly condition indices and a Pearson product–moment correlation was applied to see if there were correlations between CI and tissue weight or temperature. A Spearman rank order correlation was used to determine if there was an association between temperature and reproduction. A χ^2^ test, with the Yates correction, was utilized to ascertain if the sex ratio adhered to a 1:1 ratio.

## Results

### Temperature data

In 2010, the water temperature (recorded using ®Tidbit™) at Bannow Bay ranged from −1.66°C in December 2010 to 27.97°C in June 2010 with a yearly mean of 13.55°C. For the 6 months that the temperature probes were deployed in 2011, the mean temperature recorded was 10.74°C and it ranged from −0.44°C in January 2011 to 26.36°C in June 2011. For the whole study period, 2010 and 2011, the mean monthly temperature range in Bannow Bay was −1.66°C in December 2010 to 27.97°C in June 2010 with a mean of 12.34°C. The water temperature in 2010 at Flaxfort Strand ranged from −3.63°C in December 2010 to 26.16°C in June 2010 with a mean of 13.19°C. The mean temperature recorded from January to June 2011 was 10.53°C, the minimum value recorded was −1.78°C in January and the maximum was 27.44°C in June 2011. Overall, for the duration of the study, the minimum temperature recorded was −3.63°C in December 2010 and the maximum was 27.44°C in June 2011. There was no significant difference between the monthly average temperatures between the sites (Mann–Whitney test); however, mean monthly temperatures measured in 2010 were higher than the corresponding months in 2011.

### General cockle biology

**Figure 4:**
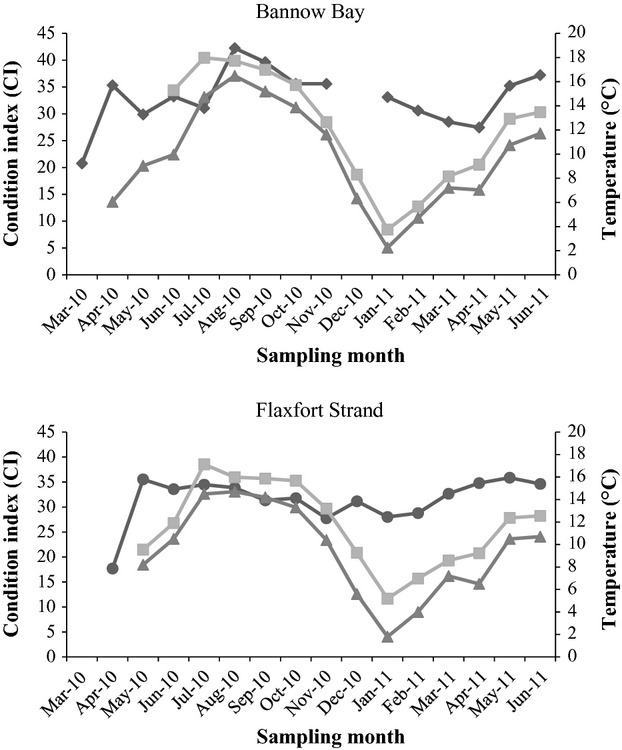
Condition index in cockles collected from Bannow Bay and Flaxfort Strand with mean monthly water and air temperature included. 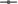, Flaxfort Strand; 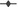, Bannow Bay; 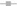, Mean Monthly Water Temperature °C; 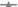, Mean Monthly Air Temperature °C.

**Figure 5:**
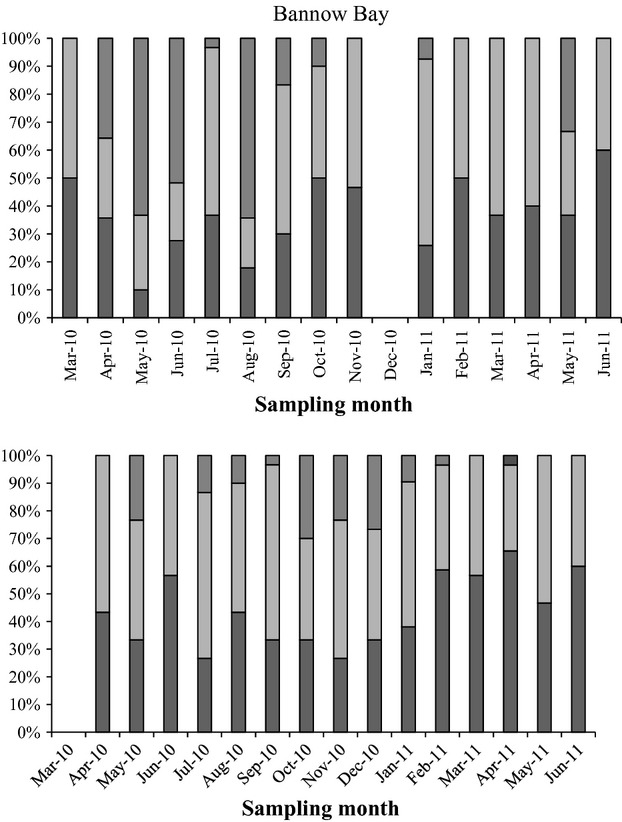
Sex ratio at both sites over the duration of the study period. 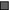, Male; 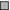, Female; 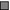, Unknown; 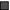, Hemaphrodite.

**Figure 6:**
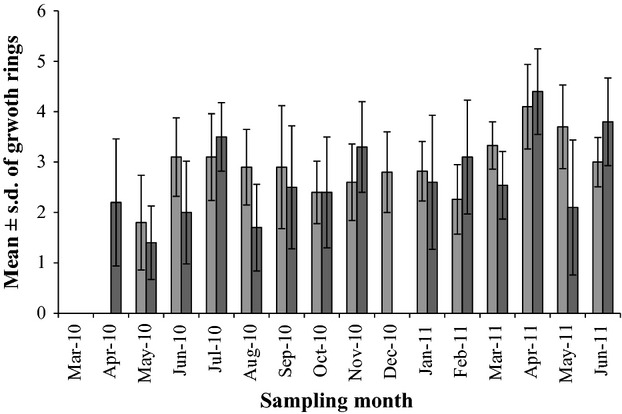
Mean ± SD age of cockles per month at Flaxfort Strand and Bannow Bay. 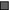, Flaxfort Strand; 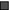, Bannow Bay.

Cockles from Bannow Bay were larger in shell length, width, and height than cockles from Flaxfort Strand, but not significantly so. However, the whole weight, shell weight, and tissue weight of cockles from Bannow Bay were significantly greater than those from Flaxfort Strand (Table 2). The mean CI of cockles collected from Bannow Bay (33.0 ± 5.28) was greater than the mean CI of cockles from Flaxfort Strand (31.5 ± 4.63), but not significantly so (Mann–Whitney test *P* = 0.436) (Fig. 4). Also, there was a greater number of fluctuations in the CI of cockles collected from Bannow Bay than Flaxfort Strand (which may be related to using wet weight rather than dry or ash-free dry weight). There was no correlation between water temperature and CI for cockles from either of the sites using a Pearson product–moment correlation. Excluding indeterminate cockles, the sex ratio at both sites did not differ significantly from a 1:1 ratio; (BB buried ♂ 163: ♀ 195: indeterminate 83: hermaphrodite 0) (FF ♂ 192: ♀ 204: indeterminate 42: hermaphrodite 1) (Fig. 5). The mean number of growth rings of cockles at Bannow Bay and Flaxfort Strand were 2.7 ± 0.84 growth rings and 2.9 ± 0.57 growth rings, respectively (Fig. 6). There was a significantly greater number of cockles with one or two growth rings at Bannow Bay (*n* = 193) than Flaxfort Strand (*n* = 138) (χ^2^ test), while the opposite occurred for three or more growth rings at (Bannow Bay *n* = 200: Flaxfort Strand *n* = 277) (χ^2^ test). At both sites, the maximum number of growth rings recorded was six.

**Table 2:** Size and weight data of *Cerastoderma edule* from both sites summarized

Measurement	Bannow Bay	Flaxfort Strand
Length (mm)	23.2 ± 5.37	22.4 ± 2.41
Range	3.2–38.1	7.2–30.2
Width (mm)	24.7 ± 5.67	24.5 ± 2.21
Range	4.1–41.1	7.6–33.3
Height (mm)	19.8 ± 6.21	18.6 ± 1.76
Range	1.5–32.9	5–29.1
Whole weight (g)	9.8 ± 4.87	7.4 ± 1.26
Range	0.02–34.16	0.28–16.5
Tissue weight (g)	2.1 ± 1.02	1.5 ± 0.30
Range	0.02–6.08	0.04–3.1
Shell weight (g)	6.4 ± 3.23	4.7 ± 0.82
Range	0.02–23.75	0.12–10.6

### Staging of gonadal maturity

Five stages of gonadal maturity were observed during the course of the study: early developing, late developing, ripe, spawning/partially spent, and spent.

### Bannow Bay

**Figure 7:**
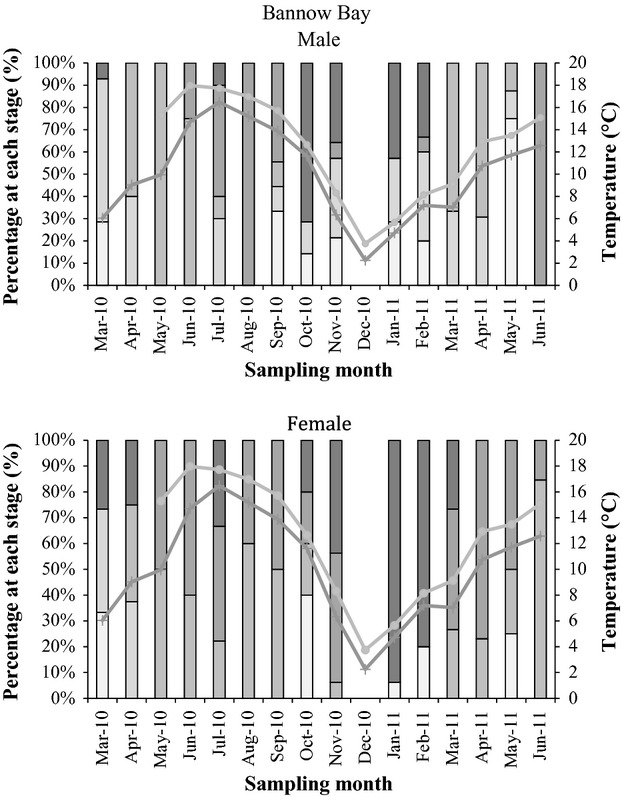
Gonadal development of male and female cockles at Bannow Bay. 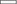, Early Developing; 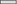, Late Developing; 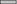, Ripe; 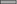, Spawning/Partially Spent; 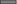, Spent; 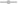, Water Temperature °C; 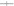, Air Temperature °C.

In March 2010 upon the commencement of the study at Bannow Bay, three stages of gonadal maturity were apparent in males: early developing (28.6%), late developing (64.3%), and spent (7.1%) (Fig. 7). Ripe males were recorded from April 2010 (60%) and spawning lasted from June 2010 (25%) until November 2010 (7.1%), 100% of males were spawning in August 2010. Gametogenesis began again in September 2010 and early developing males were seen until February 2011 when most cockles were then classified as late developing (42.9%). Ripe males were recorded from March 2011 (66.7%), but spawning did not begin until June 2011 when 100% of sampled males were spawning.

In synchrony with male cockles at Bannow Bay, females in this area were early developing (35.7%), late developing (42.9%), or spent (21.4%) in March 2010. By April 2010, ripe females (37.5%) were observed; however, some were still late developing (37.5%) and spent (25%). In May 2010, spawning began and like males continued until November 2010. Unlike males, female gametogenesis over winter was not as widespread, and gametogenesis recommenced in most female cockles in January and February 2011. It progressed rapidly to later stages of gonadal development as ripe females were identified from March 2011 (26.7%) when spawning females (46.7%) were recorded too. The peak in spawning was observed in April 2011 (76.9%) decreasing to 50% in May 2011, and by June 2011, the majority of cockles examined were in ripe condition (84.6%).

### Flaxfort Strand

**Figure 8:**
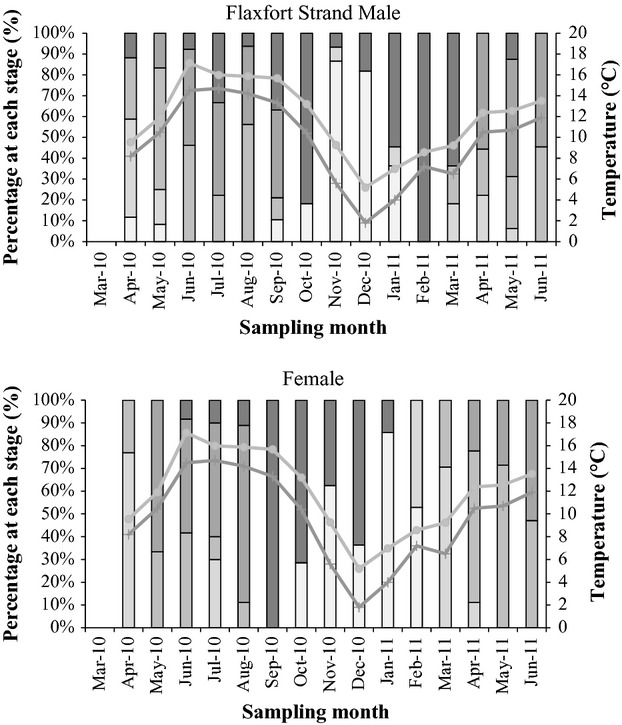
Gonadal development of male and female cockles from Flaxfort Strand. 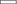, Early Developing; 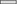, Late Developing; 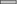, Ripe; 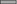, Spawning/Partially Spent; 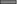, Spent; 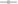, Water Temperature °C; 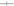, Air Temperature °C.

The study did not begin until April 2010 in Flaxfort Strand and the majority of males were late developing (76.9%), but some were ripe (23.1%) (Fig. 8). The spawning duration lasted from May 2010 until August 2010 and by September 2010, 100% of males were spent. Gametogenesis among males was visible from October 2010, but cockles developed slowly remaining as early developing until February when the later stage of gametogenesis was seen. Male cockles became ripe (29.4%) in March 2011 and spawned from April 2011 (22.2%), spawning continued until the end of the study. However, until June 2011, a greater percentage was ripe rather than spawning.

A greater range of gonadal stages was seen among females in April 2010, than in males: early developing (11.8%), late developing (47.1%), and ripe (29.4%). Spawning began in May 2010 (16.7%) and continued until September 2010 (42.1%). Gametogenesis began in September 2010 (10.5%) while other females were still ripe (10.5%) and spawning (42.1%). Development continued until more females became ripe in March 2011 (18.2%) and began spawning a month later in April 2011 (55.6%). The peak spawning time for females in 2011 was in May 2011 (56.3%) and decreased marginally to 54.5% in June 2011. Hence, spawning and its peak were a month later at Flaxfort Strand than Bannow Bay.

### Gonadal maturity and age

**Figure 9:**
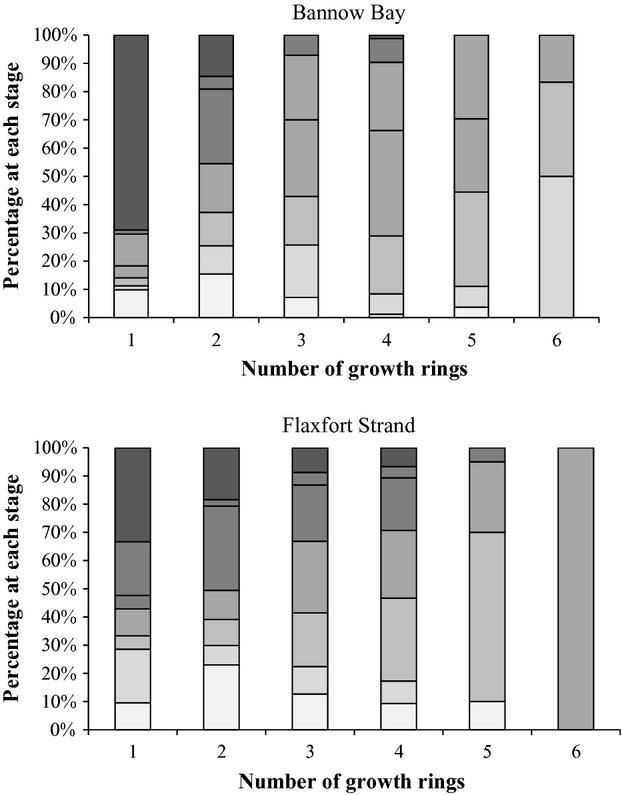
Percentage of each stage of gonadal maturity, indeterminate gonads, and no gonads per number of growth rings. 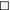, Early Developing; 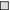, Late Developing; 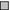, Ripe; 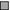, Spawning/Partially Spent; 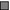, Spent; 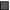, Indeterminate; 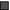, No Gonads.

The smallest shell length in which gonads were observed was 8.9 mm in Flaxfort Strand and 9.9 mm in Bannow Bay. Both were females in the early developing stage with one growth ring, but the majority of cockles with one growth ring at both sites did not develop gonads (BB 69.0%; FF 33.3%). Other stages of development were apparent in cockles with one growth ring too, these were early developing (BB 9.9%: FF 9.5%), late developing (BB 1.4%: FF 19.0%), ripe (BB 2.8%: FF 4.8%), spawning/partially spent (BB 4.2%: FF 9.5%), spent (BB 11.3%: FF 4.8%), and indeterminate (BB 1.4%: FF 19.0%). The majority (90%) of animals did not develop gonads until they were above 11 mm in length with one growth ring. At Bannow Bay, the greatest number of ripe (30.9%) and spawning (38.8%) animals had four growth rings, whereas at Flaxfort Strand, the majority had three growth rings (ripe 47.6%, spawning 59.1%) (Fig. 9).

### Gonadal maturity and temperature

Ripe gonads were seen in April 2010 at both sites in cockles when the mean monthly temperature was 9.0°C (Bannow Bay) (Fig. 7) and 9.55°C (Flaxfort Strand) (Fig. 8). Spawning was initiated in 2010 at Bannow Bay at 15.29°C (May 2010) and in Flaxfort at 11.91°C (May 2010). In the following year, ripeness and spawning began at different temperatures; ripe gonads were seen in males from Bannow Bay when the temperature was 9.13°C (March 2011) and the majority spawned at 15.09°C (June 2011), whereas females were identified as ripe and spawned at 12.93°C (April 2011). Ripening of cockle gonads in Flaxfort Strand in 2011 occurred when the mean monthly temperature was 9.23°C (March 2011) and spawning at 12.37°C (April 2011).

## Discussion

The cockle plays a significant role in soft sediments and bays where it is found in abundance and climate is one factor, mainly through temperature variation, that can impact on both reproductive capacity and recruitment, and therefore overall biomass of the bivalve. Other parameters such as competition and food availability that were not measured in this study may also impact upon reproduction and growth; however, long-term changes in climate may alter these too. Results would indicate that climate variability is impacting on reproduction of this bivalve by prolonging the period over which spawning extends. Whether in the long term, this translates into more recruitment will depend on the external factors that contribute to this process, for example, plankton blooms and how they are affected by this climate change in terms of when they occur and which particular species will dominate.

Temperatures recorded over the study period were anomalous for Ireland; the winters of 2009–2010 and 2010–2011 were exceptional for several reasons: the winter of 2009–2010 was the coldest winter since 1962–1963. Additionally, it was the sunniest winter on record and had a lower frequency of strong winds. Although the winter of 2010–2011 was noteworthy as the cold spell began earlier in November 2010 than in previous years, it also had the coldest day measured in Ireland and nine uninterrupted days during which the temperature did not rise above 0°C (http://www.npws.ie/en/SPA/004081/). Summer temperatures in 2011 were also lower than normally experienced. This period of extreme cold was in comparison to warming trends on land and sea in Europe, with the average land temperature from 2002 to 2011 being 1.3°C higher than pre-industrial levels (http://www.eea.europa.eu/data-and-maps/indicators/global-and-european-temperature/global-and-european-temperature-assessment-5). It is believed that the cold temperatures were as a result of the negative phase of the NAO, which was at its most negative value (for which there are data) continuing and North Atlantic atmospheric blocking conditions (Cattiaux et al. 2010; Sillmann et al. 2011; Vicente-Serrano et al. 2011). Whether the NAO index is positive or negative is not thought to be impacted by anthropogenic warming; however, it may influence multidecadal variability, with extended time spans of extreme positive or negative NAO index possibly becoming more common if temperature increases persist (Goodkin et al. 2008). Temperatures recorded in 2010 were higher than the corresponding months in 2011 at both sites and there were site differences in temperature. There are differences in the tidal cycle (its duration) between the sites, which could have contributed to the differences observed in temperature.

Cockles from Bannow Bay had a higher weight than cockles from Flaxfort Strand, but a greater number of cockles at Bannow Bay was younger generally, having less growth rings, indicating that overall cockles at this site grew faster than at Flaxfort Strand. The difference in cockle lengths and CI (although not significant) and weights between the two sites could be linked first to immersion duration, second the detrimental effects of prolonged emersion, and finally to the extent of macroalgal growth (de Montaudouin 1996); all of which are factors, which differ between the sites. Previous studies of cockle pathology at Flaxfort Strand have shown that the frequency of digenean trematodes and disseminated neoplasia is highest in intermediately (18.0–23.9 mm) sized cockles; so many cockles may die before attaining maximum size (Morgan et al. 2012).

There was a significantly greater number of cockles with two or less growth rings at Bannow Bay than Flaxfort Strand, which could be related to site differences in water quality. Under stressful conditions, it has been shown that another bivalve – *Mytilus edulis* produces less viable larvae (Bayne et al. 1978) and bivalve larvae themselves are highly susceptible to the effects of pollutants such as heavy metals. Flaxfort Strand, unlike Bannow Bay, is surrounded by roads and there may be run off of hydrocarbons during rains and these pollutants may affect the recruitment of cockles in this area (Beiras and His 1985). Despite having less older cockles, there are higher densities of cockles at Flaxfort Strand than at Bannow Bay (preliminary study); therefore, high densities of larvae could be filtered out of the water column before settlement.

More females were identified than males, but no significant deviation from a 1:1 ratio was observed in agreement with previous Irish and English studies (Boyden 1971; Morgan et al. 2012). A number of indeterminate cockles and one possible hermaphrodite were seen at Flaxfort Strand. Cockles of all size classes were collected, leading to the gathering of sexually immature cockles; additionally, some cockles' gonads upon examination were castrated by trematodes (pers. obs.). In all, 22% of cockles in a study from the Crouch Estuary, England were of indeterminate sex (Boyden 1971). One hermaphrodite was also found in the study on the Kent coast, but this was a mixed study of *C. edule* and *C. glaucum* and the species was not stated.

Gonads were observed in cockles of 8.9 mm in length in Bannow Bay and 9.9 mm in Flaxfort Strand or in cockles with one growth ring. Gametogenesis in cockles has previously been recorded in animals with one growth ring (Cardoso et al. 2009). However, this precocious development was rare, with most not developing gonads until >11 mm. In another study of cockle reproduction in the Dutch Wadden Sea, gonads were identified in cockles >12 mm (Cardoso et al. 2009). At Strangford Lough, initial development of gonads was recorded in cockles from 15 to 20 mm (Seed and Brown 1977). According to Dabouineau and Ponsero (2009) size, not age of cockles, could be of greater importance to cockle sexual maturity. The majority of spawning cockles had three or four growth rings in this study. This is in contrast to cockles from the Burry Inlet where precocious spawning occurred with the majority of cockles with one growth ring spawning and subsequently dying, perhaps as a reaction to high densities (Elliot et al. 2012). One or 2-year classes disproportionately contributing to the overall cockle population has been described previously in the Wadden Sea (Cardoso et al. 2009) and greater production by older cockles has also been described in the Mundaca Estuary (Iglesias and Navarro 1991).

In this study, there were differences observed between the sites and between the sexes in peaks of gonadal maturity, spawning duration, and period. Two peaks in spawning were observed at both sites, which were also previously recorded in Ireland (Morgan et al. 2012). Cockles were ripe in both sites from April 2010, but in March in 2011. Advancement of gonadal maturity was seen in male soft shell clams, *Mya arenaria* in March and April 2011 in comparison with 2010 when most males developed in May, at Bannow Bay, which was studied over the same time period (Cross et al. 2012). Ripe gonads have been described previously in cockles from May in the southeast of England (Kingston 1974) and in another study in Strangford Lough; males were described as ripe from April, but females not until May (Seed and Brown 1977). This study may be demonstrating that previous severe winters may be accelerating gametogenesis with ripe individuals being observed earlier in the year. Yankson (1986) measuring fecundity with GVF reported an earlier peak in fecundity among cockles in the Burry Inlet in 1982 after a severe winter compared to the previous year.

The spawning period extended until November in Bannow Bay, but ceased in September in Flaxfort Strand. The spawning period in intertidal cockles in the Dutch Wadden Sea continued until October (Cardoso et al. 2009), but in an earlier study in Ireland, the majority of cockles were in a spent condition from August/September (Seed and Brown 1977). Differences in spawning period could be due to site conditions, cockles at Bannow Bay were heavier than at Flaxfort Strand and there may be a greater supply of nutrients for gonad production in Bannow Bay (Cotter et al. 2010). If there is a greater amount of gonads, it may take a longer time period to spawn. It has been suggested also that reproductive strategy of a species may not be set, but could vary, with the prevailing conditions influencing the strategy adopted (Yankson 1986).

Partial spawning was observed in this study, with a low number of individuals remained partially spent over winter with residual gametes apparent; this was particularly evident at Bannow Bay. Complete spawning was found among *C. edule* in the Crouch Estuary and the Dutch Wadden Sea (Boyden 1971; Cardoso et al. 2009). Gametogenesis was seen in both sites over winter, but at both sites, the sexes were not in synchrony. Navarro et al. (1989) proposed that gametogenesis begins again over winter if sufficient glycogen reserves have been amassed during the previous summer. Rapid gonadal development initiated in spring at Strangford Lough, Northern Ireland has been described at the beginning of the growing season after over wintering in spent condition (Seed and Brown 1977; Cardoso et al. 2009); however, slow gonad development has been described in the Crouch Estuary, Essex and in the Burry Inlet (Boyden 1971; Yankson 1986). In the latter scenario, growth begins in autumn and continues over winter with increased impetus in spring and spawning commences in late spring/early summer (Boyden 1971; Yankson 1986; Twomey 1987) (Table 1).

It has been stated that in cockles, temperature does not have a coordinating effect on reproduction, either by a minimum threshold required or by temperature change (Navarro et al. 1989). However, changes in the CI of cockles from St-Pol-de-Léon, France, from 1987 to 1989, reflected alterations in temperature, with lows during the colder year of 1987 in comparison with highest CI during 1989, which was warmer than average (Guillou and Tartu 1992). Other studies indicate increases in temperature, not its absolute value, influence cockle reproduction (Dabouineau and Ponsero 2009). In this study, ripening and spawning of cockles at both sites occurred at different temperatures between years as was also observed in the Mundaca Estuary, North Spain (Navarro et al. 1989) and prevailing environmental conditions altering gametogenesis and physiology in cockles has been suggested (Iglesias and Navarro 1991). Gonadal maturity could be attributed to not only temperature but also the animal's condition and food availability (Yan et al. 2010). This study coincided with two severe winters, anomalous to Ireland (http://www.epa.ie/downloads/pubs/water/waterqua/waterrep/). Particularly, cold winters may additionally aid reproductive success in cockles by ensuring that male and female gametes are released in unison (Dabouineau and Ponsero 2009). A study of cockle gametogenesis in the Burry Inlet in 1981–1982 corresponded as well with an unusually cold winter. Their findings (Yankson 1986) included incomplete spawning, followed by redevelopment and spawning again, which was in agreement with the incomplete spawning seen in this study.

Milder winters experienced in the Wadden Sea area have been linked to on-going reductions in bivalve recruitment (Philippart et al. 2003), whereas several studies have reported greater bivalve recruitment after severe winters (Honkoop et al. 1998; Philippart et al. 2003; Beukema and Dekker 2005). Less severe milder winters are thought to allow for gametogenesis to occur for a longer period, therefore, not allowing for a cessation in gonad production and a rest period for the bivalve, but colder winters provide a rest period between cycles, which may be advantageous for redevelopment in spring (Guillou and Tartu 1992). Levels of interannual variation can be observed nonetheless in many marine invertebrates and this phenomenon is not totally understood (Honkoop et al. 1998). Direct (variation in egg numbers) and indirect (predator–prey mismatch) effects of climate change are thought to be responsible for this occurrence (Flach 2003). Cardoso et al. (2009) believe that *C. edule* may be more affected by climate change than other more long-lived bivalves such as *Mya arenaria* as the possibility of them experiencing a cold winter during their short lifespan is low.

Moore et al. (2011) postulate that comparing historical and contemporary data has limitations, but may be informative in relation to species responses to climate change in particular, if baseline data are absent. Although different methods of assessment were used, it appears that the spawning period of cockles has extended throughout the last century (Table 3), with an earlier starting time and continuing later into autumn. This could be linked to the change in environmental conditions, orchestrated by global climate change, with globally oceans warming on average by 0.6°C in the last 100 years (Wiltshire et al. 2008) and European seas are thought to be especially affected by climate change (Philippart et al. 2011). Alterations in phenological relationships due to sea temperature will have consequences modifying many trophic interactions such as food webs (Edwards and Richardson 2004; Wiltshire et al. 2008). Indeed, marine temperate areas could be especially susceptible to changes in the cycle of plankton production as recruitment is contingent upon it and spring plankton blooms are occurring earlier (Edwards and Richardson 2004; Heath et al. 2012). Therefore, earlier spawning in cockles and longer spawning period could be as a consequence of amendments to plankton pulses. Extension of the breeding season could reduce the chances of a mismatch with peaks in primary production and prevent the chances of overpopulation by mass spawning, as has been described in *Cerastoderma glaucum* (Guillou and Tartu 1992). This could be exacerbated in cockles, as previously stated, as income breeders; they can quickly utilize food resources for gonad manufacture (Cardoso et al. 2009). Other invertebrates such as the brown shrimp *Crangon crangon* have altered their spawning regime in response to warmer temperatures by having two spawning periods in the southern North Sea, compared with one in the northern North Sea (Heath et al. 2012). Similarly, the boreal limpet *Patella vulgata* spawns further into the autumn in the 2000s than it did during the 1940s (Moore et al. 2011).

**Table 3:** Previous study locations and methods of assessing *Cerastoderma edule* gametogenesis arranged by latitude

Location	Method	Gametogenesis	Spawning Period	Reference
Trondheim, Norway	Gonad smears		Early July began	Rygg 1970;
Western Wadden Sea	Gonadal mass ratio		April–October	Cardoso et al. 2009;
Strangford Lough, Ireland	Gonad classification	Spring	Midsummer–August	Seed and Brown 1977;
Cardigan Bay, Wales	Histology	October–March	May began	Creek 1960;
Crouch Estuary, England	Gonadal smears	Early Spring	May–July	Boyden 1971;
Burry Inlet, Wales	Gonad smears		June began	Hancock and Franklin 1972;
Kent Coast, England	Histology	September and October	May and June	Kingston 1974;
Plymouth, England	Sieving		May–August	Lebour 1938;
Mundaca Estuay, Basque Country, Spain	Gamete volume fraction	November	May–September	Navarro et al. 1989

In spite of trends in the Wadden Sea and western Sweden toward milder winters, this study was conducted during a time of uncharacteristically cold winters in Ireland, which appears to bode well for cockle recruitment success. However, this was followed by a mild winter in 2011–2012, so it is difficult to predict future winter conditions. Although previously in Ireland gametogenesis has been described from spring only, this study found that cockles begin to develop gonads over winter. The duration of spawning time reported in this study also contrasts with previous studies, starting earlier and continuing until later in autumn. Males and females in both sites, additionally, were not entirely synchronous in terms of gametogenesis and spawning, which if the time span widens could impact negatively upon spawning success (for a summary of the main results refer to Table 4). The lack of younger year classes of cockles present at Flaxfort Strand could have serious ramifications for the many birds that rely upon them as a food source. Particularly, birds classified as Annex 1 on the EU Birds Directive, for example, bar-tailed godwits and golden plovers frequent this area.

**Table 4:** Summary of main results

Temperature	No significant difference between sites, but 2010 was warmer than the corresponding months in 2011.
General cockle biology	Cockles from Bannow Bay greater in length, width, and shell height (but not significantly so).
	Significantly greater whole weight, shell weight, and tissue weight in Bannow Bay.
	Greater condition index at Bannow Bay (but not significantly so).
	Sex ratio adhered to 1:1, but females were more common at both sites.
	Significantly more young cockles (with less than two growth rings) at Bannow Bay
Reproduction	Five stages of gonadal maturity identified.
	After a severe winter, gonad ripening and spawning began earlier.
	Ripening and spawning were recorded at different temperatures.
	The smallest cockle with gonads was 8.9 mm (Flaxfort Strand) and 9.9 mm (Bannow Bay).
	Most cockles (90%) did not develop gonads until they were above 11 mm in length.
	Cockle gametogenesis appears to have extended in duration, especially when compared with other studies.
	Gametogenesis began over winter, in contrast to previous study in Ireland.

Alterations in cockle gametogenesis, spawning and subsequent settlement are not only dependent on temperature but also the effect that climate variability has on plankton and previously appropriate habitats may no longer be suitable due to changes in oxygen levels or thermal regime. Reductions in cockle numbers would have concurrent effects on water chemistry, sediment oxygenation, and structure and the species richness of an area. Further studies using other intertidal organisms such as mussels or barnacles could be beneficial, to see if changes in spawning or larval release timing is widespread in marine shallow water invertebrates.

## References

[b1] André C, Rosenberg R (1991). Adult-larval interactions in the suspension-feeding bivalves *Cerastoderma edule* and *Mya arenaria*. Mar. Ecol. Prog. Ser.

[b2] André C, Lindegarth M, Jonsson PR, Sundberg P (1999). Species identification of bivalve larvae using random amplified polymorphic DNA (RAPD): differentiation between *Cerastoderma edule* and *C. lamarki*. J. Mar. Biol. Assoc. U. K.

[b3] Bayne BL, Holland DL, Moore NM, Lowe DM, Widdows J (1978). Further studies on the effects of stress in the adult on the eggs of *Mytilus edulis*. J. Mar. Biol. Assoc. U. K.

[b4] Beiras R, His E (1985). Effects of dissolved mercury on embryogenesis, survival and growth of *Mytilus galloprovincialis* mussel larvae. Mar. Ecol. Prog. Ser.

[b5] Belkin LM (2009). Rapid warming of large marine ecosystems. Prog. Oceanogr.

[b6] Beukema JJ, Dekker R (2005). Decline of recruitment success in cockles and other bivalves in the Wadden Sea: possible role of climate change, predation on postlarvae and fisheries. Mar. Ecol. Prog. Ser.

[b7] Bouma H, Duiker JMC, Herman PP, de Vries PMJ, Wolff WJ (2001). Spatial pattern of early recruitment of *Macoma balthica* (L.) and *Cerastoderma edule* (L.) in relation to sediment dynamics on a highly dynamic intertidal sandflat. J. Sea Res.

[b8] Boyden CR (1971). A comparative study of the reproductive cycles of the cockles *Cerastoderma edule* and *C. glaucum*. J. Mar. Biol. Assoc. U. K.

[b9] Brown RA (1984). Geographical variations in the reproduction of the horse mussel, *Modiolus modiolus* (Mollusca: Bivalvia). J. Mar. Biol. Assoc. U.K.

[b10] Cardoso JFMF, Witte JI, van der Veer HW (2009). Differential reproductive strategies of two bivalves in the Dutch Wadden Sea. Estuar. Coast. Shelf Sci.

[b11] Cattiaux JR, Vautard R, Cassou C, Yiou P, Masson-Delmotte V, Codron F (2010). Winter 2010 in Europe: a cold extreme in a warming climate. Geophys. Res. Lett.

[b12] Cesar CP (2009). The roles of the cockle Cerastoderma edule L. on ecosystem functioning: cockle comings and goings.

[b13] Cotter E, Malham SK, O'Keefe S, Lynch SA, Latchford JW, King JW (2010). Summer mortality of the Pacific oyster, *Crassostrea gigas*, in the Irish Sea: the influence of growth, biochemistry and gametogenesis. Aquaculture.

[b14] Creek GA (1960). Development of the lamellibranch *Cardium edule* (L.). J. Zool.

[b15] Cross ME, Lynch S, Whitaker A, O'Riordan RM, Culloty SC (2012). The reproductive biology of the softshell clam, *Mya arenaria*, in Ireland, and the possible impacts of climate variability. J. Mar. Biol.

[b16] Dabouineau L, Ponsero A (2009). Synthesis on biology of the common European cockle Cerastoderma edule.

[b17] Drummond L, Mulcahy M, Culloty SC (2006). The reproductive biology of the Manila clam, *Ruditapes philippinarum*, from the North-West of Ireland. Aquaculture.

[b18] Edwards M, Richardson AJ (2004). Impact of climate change on marine pelagic phenology and trophic mismatch. Nature.

[b19] Elliot M, Burdon D, Callaway R (2012). Burry Inlet cockle mortalities investigation 2009–2011. https://publications.environment-agency.gov.uk/skeleton/publications/SearchResults.aspx?name=GEWA0112BWAP-E-E.

[b20] Flach EC (2003). The separate and combined effects of epibenthic predation and presence of macro-infauna on the recruitment success of bivalves in shallow soft sediment areas on the Swedish west coast. J. Sea Res.

[b21] Frost M, Baxter JM, Buckley PJ, Cox M, Dye SR, Withers Harvey N (2012). Impacts of climate change on fish, fisheries and aquaculture. Aquat. Conserv. Mar. Freshw. Ecosyst.

[b22] Goodkin NF, Hughen KA, Doney SC, Curry WB (2008). Increased multidecadal variability of the North Atlantic Oscillation since 1781. Nat. Geosci.

[b23] Gosling EM (2003). Bivalve molluscs: biology, ecology and culture.

[b24] Guillou J, Tartu C (1992). Reproduction et recrutement de la coque Cerastoderma edule.

[b25] Guillou J, Tartu C (1994). Post-larval and juvenile mortality in a population of the edible cockle *Cerastoderma edule* (L.) from Northern Brittany. Neth. J. Sea Res.

[b26] Hancock DA, Franklin A (1972). Seasonal changes in the condition of the edible cockle *Cardium edule* (L.). J. Appl. Ecol.

[b27] Heath MR, Neat FC, Ponnegar JK, Reid DG, Sims DW, Wright PJ (2012). Review of climate change impacts on marine fish and shellfish around the UK and Ireland. Aquat. Conserv. Mar. Freshw. Ecosyst.

[b28] Honkoop PJC, van der Meer J (1998). Experimentally induced effects of water temperature and immersion time on reproductive output of bivalves in the Wadden Sea. J. Exp. Mar. Biol. Ecol.

[b29] Honkoop PJC, Beukema J, van der Meer JJ, Kwast D (1998). Does temperature-influenced egg production predict the recruitment in the bivalve *Macoma balthica*. Mar. Ecol. Prog. Ser.

[b30] Howard DW, Smith CS (1983). NOAA Technical Memorandum NMFS -F/ NEC 25. Histological Techniques for Marine Bivalve Mollusks.

[b31] Iglesias JIP, Navarro E (1991). Energetics of growth and reproduction in cockles (*Cerastoderma edule*): seasonal and age-dependant variations. Mar. Biol.

[b32] Kingston PF (1974). Studies on the reproductive cycles of *Cardium edule* and *C. glaucum*. Mar. Biol.

[b33] Lebour MV (1938). Notes on the breeding of some lamellibranchs from Plymouth and their larvae. J. Mar. Biol. Assoc. U. K.

[b34] Lindegarth M, André C, Jonsson PR (1995). Analysis of the spatial variability in abundance and age structure of two infaunal bivalves, *Cerastoderma edule* and *C. lamarcki*, using hierarchical sampling programs. Mar. Ecol. Prog. Ser.

[b35] Madeira D, Narciso L, Cabral HN, Vinagre C (2012). Thermal tolerance and potential impacts of climate change on coastal and estuarine organisms. J. Sea Res.

[b36] de Montaudouin X (1996). Factors involved in growth plasticity of cockles *Cerastoderma edule* (L.), identified by field survey and transplant experiments. J. Sea Res.

[b37] de Montaudouin X, Thieltges D, Gam M (2009). Digenean trematode species in the cockle *Cerastoderma edule*: identification key and distribution along the north-eastern Atlantic shoreline. J. Mar. Biol. Assoc. U. K.

[b38] Moore PJ, Thompson RC, Hawkins SJ (2011). Phenological changes in intertidal con-specific gastropods in response to climate warming. Glob. Change Biol.

[b39] Morgan E, O'Riordan RM, Kelly TC, Culloty SC (2012). Influence of disseminated neoplasia, trematode infections and gametogenesis on surfacing and mortality in the cockle *Cerastoderma edule*. Dis. Aquat. Org.

[b40] Navarro E, Iglesias JIP, Larranaga A (1989). Interannual variation in the reproductive cycle and biochemical composition of the cockle *Cerastoderma edule* from the Mundaca Estuary (Biscay, North Spain). Mar. Biol.

[b41] Philippart CJM, Beukema HM, van Aken JJ, Bos OG, Cadée GC, Dekker R (2003). Climate-related changes in recruitment of the bivalve *Macoma balthica*. Limnol. Oceanogr.

[b42] Philippart CJM, Anadón R, Danovaro R (2011). Impact of climate change on European marine ecosystems: observations, expectations and indicators. J. Exp. Mar. Biol. Ecol.

[b43] Ramόn M (1996). Relationships between the bivalves *Mytilus edulis* L. and *Cerastoderma edule* L. in a soft bottom environment: an example of interaction at small spatial scale. J. Exp. Mar. Biol. Ecol.

[b44] Reise K (2003). Metapopulation structure in the lagoon cockle *Cerastoderma lamarki* in the northern Wadden Sea. Helgol. Mar. Res.

[b45] Rygg B (1970). Studies on *Cerastoderma edule* (L.) and *Cerastoderma glaucum* (Poiret). Sarsia.

[b46] Sauriau P-G, Kang C-K (2000). Stable isotope evidence of benthic microalgae based growth and secondary production in the suspension feeder *Cerastoderma edule* (Mollusca, Bivalvia) in the Marennes-Oleron Bay. Hydrobiologia.

[b47] Schiedek D, Sundelin B, Readman JW, Macdonald RW (2007). Interactions between climate change and contaminants. Mar. Pollut. Bull.

[b48] Seed R, Brown RA (1977). A comparison of the reproductive cycles of *Modiolus modiolus* (L.), *Cerastoderma**Cardium**edule* (L.), and *Mytilus edulis* L. [sic] in Strangford Lough, Northern Ireland. Oecologia.

[b49] Shaw BL, Battle HI (1957). The gross and microscopic anatomy of the digestive tract of the oyster, *Crassostrea virginica* (Gmelin). Can. J. Zool.

[b50] Sillmann J, Croci-Maspoli M, Kallache M, Katz RW (2011). Extreme cold winter temperatures in Europe under the influence of North Atlantic atmospheric blocking. J. Clim.

[b51] Strasser M, Reinwald T, Reise K (2001). Differential effects of the severe winter of 1995/96 on the intertidal bivalves *Mytilus edulis*
*Cerastoderma edule* and *Mya arenaria* in the Northern Wadden Sea. Helgol. Mar. Res.

[b52] Twomey E (1987). A sarcoma of the cockle Cerastoderma edule.

[b53] Tyler-Walters H (2007). Cerastoderma edule. Common COCKLE. Marine life information network: biology and sensitivity key information sub-programme [on-line].

[b54] UKMMAS – United Kingdom Marine Monitoring & Assessment Strategy (2010). http://chartingprogress.defra.gov.uk/charting-progress2005.

[b55] Vicente-Serrano SM, Trigo RM, López-Moreno JI, Liberato MLR, Lorenzo-Lacruz J, Beguería S (2011). Extreme winter precipitation in the Iberian Peninsula in 2010: anomalies, driving mechanisms and future projections. Clim. Res.

[b56] Wiltshire KH, Malzahn AM, Wirtz K (2008). Resilience of North Sea phytoplankton spring bloom dynamics: an analysis of long-term data at Helgoland Roads. Limnol. Oceanogr.

[b57] Yan H, Li Q, Liu W, Yu R, Kong L (2010). Seasonal changes in reproductive activity and biochemical composition of the razor clam *Sinonovacula constricta* (Lamark 1818). Mar. Biol. Res.

[b58] Yankson K (1986). Reproductive cycles of *Cerastoderma glaucum* (Bruguière) and *C. edule* (L.) with special reference to the effects of the severe 1981–1982 Winter. J. Mollusc. Stud.

